# Childhood obesity treatment; Effects on BMI SDS, body composition, and fasting plasma lipid concentrations

**DOI:** 10.1371/journal.pone.0190576

**Published:** 2018-02-14

**Authors:** Tenna Ruest Haarmark Nielsen, Cilius Esmann Fonvig, Maria Dahl, Pernille Maria Mollerup, Ulrik Lausten-Thomsen, Oluf Pedersen, Torben Hansen, Jens-Christian Holm

**Affiliations:** 1 The Children’s Obesity Clinic, Department of Pediatrics, Copenhagen University Hospital Holbæk, Holbæk, Denmark; 2 Novo Nordisk Foundation Center for Basic Metabolic Research, Faculty of Health and Medical Sciences, University of Copenhagen, Copenhagen, Denmark; 3 Hans Christian Andersen Children's Hospital, Odense University Hospital, Odense, Denmark; 4 Department of Clinical Medicine, University of Copenhagen, Copenhagen, Denmark; GeneDx, UNITED STATES

## Abstract

**Objective:**

The body mass index (BMI) standard deviation score (SDS) may not adequately reflect changes in fat mass during childhood obesity treatment. This study aimed to investigate associations between BMI SDS, body composition, and fasting plasma lipid concentrations at baseline and during childhood obesity treatment.

**Methods:**

876 children and adolescents (498 girls) with overweight/obesity, median age 11.2 years (range 1.6–21.7), and median BMI SDS 2.8 (range 1.3–5.7) were enrolled in a multidisciplinary outpatient treatment program and followed for a median of 1.8 years (range 0.4–7.4). Height and weight, body composition measured by dual-energy X-ray absorptiometry, and fasting plasma lipid concentrations were assessed at baseline and at follow-up. Lipid concentrations (total cholesterol (TC), low-density lipoprotein (LDL), high-density lipoprotein (HDL), non-HDL, and triglycerides (TG)) were available in 469 individuals (264 girls). Linear regressions were performed to investigate the associations between BMI SDS, body composition indices, and lipid concentrations.

**Results:**

At baseline, BMI SDS was negatively associated with concentrations of HDL (*p* = 6.7*10^−4^) and positively with TG (*p* = 9.7*10^−6^). Reductions in BMI SDS were associated with reductions in total body fat percentage (*p*<2*10^−16^) and percent truncal body fat (*p*<2*10^−16^). Furthermore, reductions in BMI SDS were associated with improvements in concentrations of TC, LDL, HDL, non-HDL, LDL/HDL-ratio, and TG (all p <0.0001). Changes in body fat percentage seemed to mediate the changes in plasma concentrations of TC, LDL, and non-HDL, but could not alone explain the changes in HDL, LDL/HDL-ratio or TG. Among 81 individuals with available lipid concentrations, who increased their BMI SDS, 61% improved their body composition, and 80% improved their lipid concentrations.

**Conclusion:**

Reductions in the degree of obesity during multidisciplinary childhood obesity treatment are accompanied by improvements in body composition and fasting plasma lipid concentrations. Even in individuals increasing their BMI SDS, body composition and lipid concentrations may improve.

## Introduction

Childhood obesity is a major and rapidly growing public health challenge worldwide [1]. Obesity is defined as excess body fat [2] and, although various methods for estimation hereof exist, dual-energy X-ray absorptiometry (DXA) is considered a valid and clinically applicable method [3,4]. Body mass index (BMI) is often used in pediatrics, as it is a readily accessible measure of body fatness, and has been shown to exhibit robust associations with body fat measured by DXA in 198 healthy Italian children and adolescents [5]. However, as BMI has a low sensitivity (66%) and a high specificity (94%) as a fatness measure, individuals with overweight and obesity may wrongly be classified as normal weight [6,7]. Hence, in a pediatric context, DXA scans are ideally used for obesity evaluation [8]. Body fat distribution is influenced by i.e. sex, age, and ethnicity and may vary considerably in subjects with similar BMI [9,10]. In children and adolescents, this variability is more pronounced between the 85^th^ and 94^th^ percentile for BMI than for BMI below the 85^th^ percentile [11]. Furthermore, an increased central fat deposition is associated with increased metabolic complications including increased lipid concentrations [12]. In healthy individuals with normal weight, excess visceral fat and impaired lipid metabolism still constitutes an increased risk of developing the metabolic syndrome and cardiovascular disease (CVD). In these individuals, a decrease in lean mass was associated with higher CVD risk, indicating that decreased lean mass, rather than increased fat mass, may predict CVD [13].

We have previously, in 240 children and adolescents, demonstrated that weight loss during multidisciplinary and multifaceted obesity treatment improves fasting plasma concentrations of total cholesterol (TC), LDL, HDL, non-HDL, and triglycerides (TG) in children with overweight or obesity [14]. Reductions in BMI standard deviation score (SDS) were associated with improvements of fasting plasma lipids, however, some children increased their BMI SDS while improving their fasting plasma lipid profile [14]. Thus, we hypothesized that this was due to favorable changes in body composition, not reflected by reductions in BMI SDS. Hence, the aim of the present study was to investigate baseline associations between BMI SDS, body composition, and fasting plasma lipid concentrations, and to investigate whether changes in body composition measured by DXA scans were associated with changes in BMI SDS, whether changes in body composition were associated with changes in fasting plasma lipid concentrations, and if changes in body composition could explain the associations between changes in BMI SDS and fasting plasma lipid concentrations.

## Patients and methods

### Study population

Patients were included from The Children’s Obesity Clinic, Department of Pediatrics at Copenhagen University Hospital Holbæk from October 2009 until December 2015.

**Fig 1:**
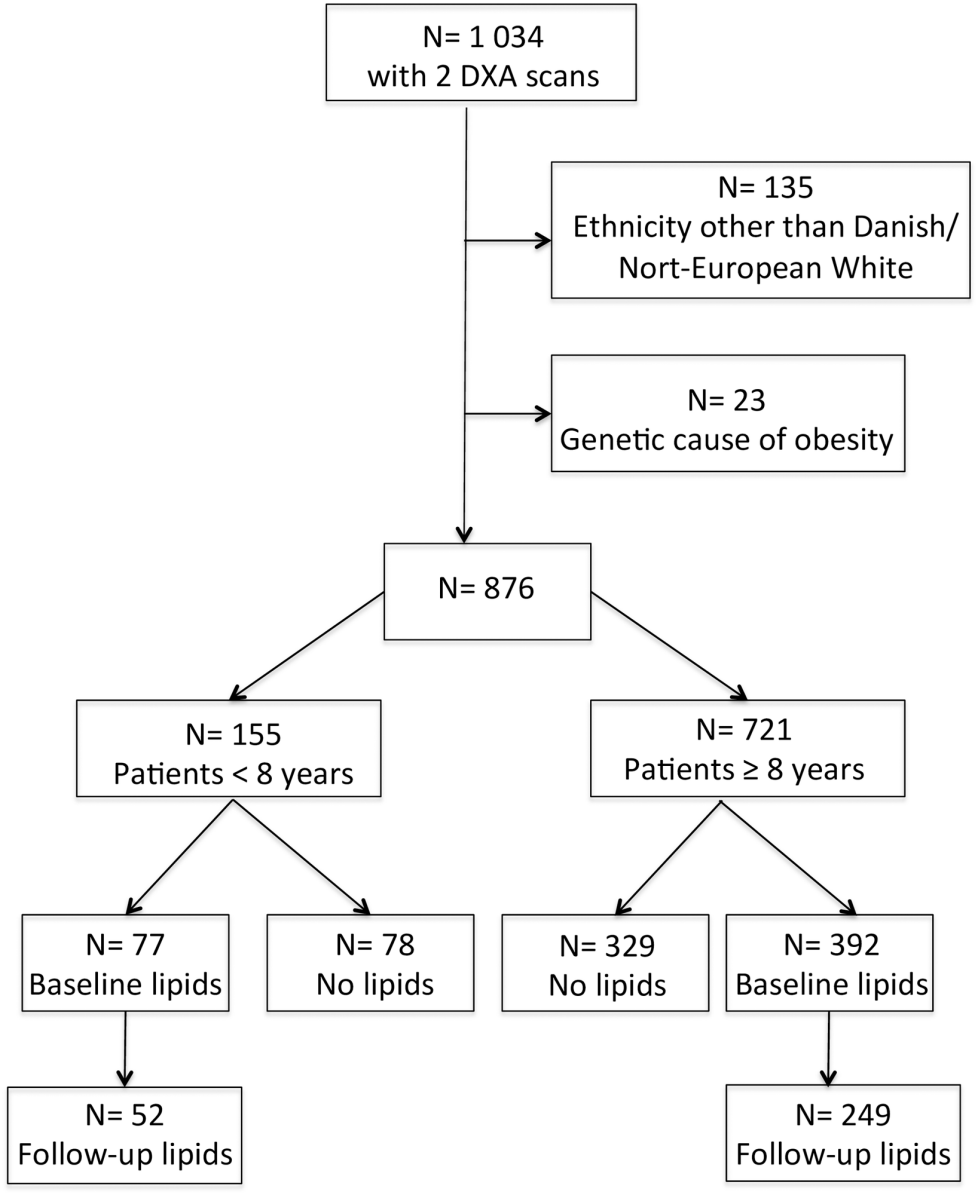
Flow-chart showing the patients in the study.

The criteria for enrollment into treatment were an age of 0–24 years and a BMI above the 90^th^ percentile for age and sex [15]. The clinic has no exclusion criteria. Children were eligible for the present study if they had a least two DXA scans performed; one at entry into treatment and one at follow-up. Patients with ethnicities other than Danish/North-European white (N = 135) or a known genetic cause of obesity (N = 23) were excluded (Fig 1).

All patients gave informed assent, and written informed consent was obtained from all parents/caretakers of children younger than 18 years of age, and from patients 18 years of age or older. The study was approved by the Regional Ethical Committee of Region Zealand (Project number: SJ-104) and the Danish Data Protection Agency, and registered at ClinicalTrials.gov (NCT00928473).

### Anthropometry

Trained medical personnel examined all patients at entry into treatment and at follow-up visits during treatment. Height was measured using a stadiometer to the nearest millimeter, and weight was measured to the nearest 0.1 kilogram, on a Soehnle Digital Indicator (Professional 2755, Soehnle, Backnang, Germany). Measurements were performed wearing light indoor clothes and without shoes.

BMI was calculated as the weight in kilograms divided by the height in meters squared. The LMS method [16] was used to calculate BMI standard deviation scores (SDS) according to a Danish reference [15] using the median (M), the coefficient of variation (S), and a measure of the skewness (L) in a Box-Cox transformation to normalize the data as described by Cole et al [17]. This was performed in order to report on the entire cohort of children and adolescents of varying age and sex, both of which influences the normal BMI distribution in children and adolescents [18].

### DXA scans

Whole body DXA scans were performed on all included patients at The Children’s Obesity Clinic, and repeated every one to two years to monitor treatment response. DXA scans were performed on a GE Lunar Prodigy (DF+10031, GE Healthcare, Madison, Wisconsin, USA) (N_baseline_ = 263, N_follow-up_ = 2) until October 14^th^ 2009, and thereafter on a GE Lunar iDXA (ME+200179, GE Healthcare, Madison, Wisconsin, USA) (N_baseline_ = 613, N_follow-up_ = 874). Daily calibration was performed to ensure that the devices were operating within the manufacturer’s specifications. Total body fat mass (BF), total lean mass (TLM), truncal fat mass, and truncal lean mass were measured. Total body fat percentage (%BF) was calculated as (BF/(total body mass—total bone mass)*100). Fat free mass index (FFMI) was calculated as TLM/height squared. The LMS method [17] was used to calculate standard deviation scores (SDS) for FFMI and %BF on all children and youths eight years of age and older based on the NHANES material recently modified to apply to the GE Lunar systems (Prodigy and iDXA) [19]. As the LMS data were only available for 8–20-year-olds, participants younger than eight years of age were excluded from the analyses including FFMI SDS and %BF SDS (N = 155). Whole body DXA can measure regional fat distribution as well [20]. As a measure of central fat accumulation the truncal body fat percentage (%TBF) was calculated as truncal fat mass/(truncal fat mass + truncal lean mass) [12].

### Blood sampling

Blood sampling was performed from 7–9 AM after an overnight fast at entry into treatment, and repeated every one to two years to monitor treatment response, in adherence to the standard operating procedure at The Children’s Obesity Clinic. In the present study, blood samples were included if they were drawn within a period of 30 days before or after the DXA scan (Fig 1). The samples were processed within one hour, and analyzed at the Department of Clinical Biochemistry, Copenhagen University Hospital Holbæk. Plasma concentrations of TC, HDL, and TG were, prior to May 16^th^ 2013, analyzed on a Cobas^®^ 6000 analyzer (Roche Diagnostics, Manheim, Germany) (N_baseline_ = 359, N_follow-up_ = 130), and on a Dimension Vista^®^1500 (Siemens Healthcare, Erlangen, Germany) (N_baseline_ = 110, N_follow-up_ = 256) from May 16^th^ 2013 onwards, due to method change at the laboratory for administrative reasons not related to this study.

Detection limits on the Cobas^®^ 6000 were 0.1 mmol/L for TG and TC, and 0.08 mmol/L for HDL, and on the Dimension Vista^®^1500, 0.02 mmol/L for TG, 1.29 mmol/L for TC, and 0.05 mmol/L for HDL according to the manufacturer. Internal quality control provided intra-assay coefficients of variations of 0.010–0.026 for TC, 0.008–0.034 for HDL, and 0.009–0.024 for TG on the Cobas^®^ 6000, and of 0.014–0.028 for TC, 0.027–0.043 for HDL, and 0.010–0.020 for TG on the Dimension Vista^®^1500.

To ensure comparability of the analyses performed on the Cobas^®^ 6000 and Dimension Vista^®^1500, routine laboratory data were extracted from the laboratory information system. The TC, HDL, and TG concentrations differed by a mean of 0.26 mmol/L, 0.06 mmol/L, and 0.08 mmol/L, respectively, when using the Dimension Vista^®^1500 versus the Cobas^®^ 6000. To account for these differences in measurement results, TC and HDL concentrations measured with the Cobas^®^ 6000 were deducted by 0.26 mmol/L and 0.06 mmol/L, respectively [21]. TG concentrations measured on Cobas^®^ 6000 were multiplied by 1.17 and deducted by 0.16 mmol/L. The Friedewald Formula [22] was used to calculate the LDL, if TG <4.5mmol/L, based on the adjusted concentrations.

### Intervention

The Children’s Obesity Clinic at the Department of Pediatrics, Copenhagen University Hospital Holbæk offers a multidisciplinary and multifaceted treatment to children and adolescents with overweight or obesity. The intervention strategy is based on the understanding of obesity as a chronic disease [23–26], where the fat mass is endocrinologically regulated, with the capacity to counteract weight loss and preserve the fat mass [27]. The treatment plan is based on the information obtained during the initial interview and physical examination, is individual and addresses all unhealthy habits in the every day life of the family from the first day. Typically 15–25 items are on the treatment plan for the child and family to follow. Items on the treatment plan includes, but is not limited to, instructions on what to eat for breakfast, lunch, dinner and in-between snacks in addition to what not to eat, instructions to drink water not soft drinks, agreements on exercise, bed times, allowances, as well as reducing daily screen time to 2 hours or less. An example of an individual treatment plan is given in S1 Table. At subsequent visits, the plan is evaluated and difficulties explored. Each child/adolescent is seen with 6–8-week intervals on average, and with an invested 5.4 hours of health professional time per patient per year [28,29]. The treatment protocol has been described in detail previously [28,29].

### Statistical analyses

Statistical analyses were performed using the statistical software R (version 3.2.4) [30]. The Wilcoxon signed rank test was used to analyze differences in baseline values of age, BMI SDS, and body composition indices between subgroups of patients with and without follow-up blood samples. Pearson’s Chi-squared test was used to investigate differences in gender distribution among subgroups and in proportions of individuals improving body composition between subgroups. Analyses of changes from baseline to follow-up within subgroups were calculated using the *t* test for paired data. Analyses of differences in changes from baseline to follow-up between subgroups were calculated using the *t* test for unpaired data.

Baseline associations between BMI SDS and body composition indices (%BF, %BF SDS, FFMI, FFMI SDS, and %TBF) measured by DXA, and fasting plasma lipid concentrations were analyzed using linear regression models adjusted for age and sex. Associations between changes in BMI SDS, body composition indices, and fasting plasma lipid concentrations were investigated using linear regression models adjusted for age at baseline, sex, baseline values of the two dependent variables, and the treatment duration. To investigate whether %BF SDS and/or FFMI SDS might mediate associations between BMI SDS and fasting plasma lipids, we further adjusted the linear regressions investigating associations between BMI SDS and fasting plasma lipids for %BF SDS or FFMI SDS, and similarly adjusted the analyses of changes for the change in %BF SDS or change in FFMI SDS. The assumption of a normal distribution was examined using qq-plots and histograms. Baseline TG and LDL/HDL-ratio were logarithmically transformed prior to linear regression analyses to achieve a normal distribution of residuals. Co-linearity of BMI SDS and body composition variables was investigated using the *vif* function in the *car* package in R. There were no issues of co-linearity in any of the linear regression models investigating associations between BMI SDS and body composition variables. Due to the change of DXA scanner method, all analyses involving %BF, %BF SDS, FFMI, FFMI SDS, and %TBF were adjusted for scanner type. The Bonferroni method was used for correction in the 36 baseline regression analyses and the 36 follow-up regression analyses of body composition indices and lipid concentrations, and the significance level of these analyses was thus set to *p*<0.0014.

## Results

876 children and adolescents age 1–22 years with a median BMI SDS of 2.76 were available for the present study after exclusions (Table 1).

**Table 1:** Characteristics at baseline and at follow-up of the patients included in the study.

	Baseline		Follow-up	
	N		N	
Age	876	11.2	876	13.5
(Years)		(1.6–21.7)		(4.4–23.2)
BMI SDS	876	2.76	876	2.5
		(1.32–5.70)		(-0.49–5.2)
%BF SDS	721	1.40	721	1.2
		(0.01–2.70)		(-1.90–2.70)
FFMI SDS	721	1.12	721	1.10
		(-2.24–4.40)		(-1.79–3.90)
%BF	876	43.2	876	41.4
		(24.6–59.3)		(17.4–60.5)
FFMI	876	13.8	876	15.0
		(10.4–24.9)		(10.4–25.8)
%TBF	876	43.5	876	42.2
		(18.4–62.5)		(12.5–62.7)
TC (mmol/L)	469	3.9	301	3.8
		(1.7–6.5)		(1.7–7.8)
LDL (mmol/L)	466	2.3	301	2.1
		(0.6–4.6)		(0.5–4.3)
HDL (mmol/L)	469	1.1	301	1.2
		(0.6–2.3)		(0.6–2.3)
TG (mmol/L)	469	0.9	301	0.8
		(0.1–4.9)		(0.2–4.6)
Non-HDL (mmol/L)	469	2.7	301	2.5
		(0.7–5.5)		(0.7–6.9)
LDL/HDL	466	1.9	301	1.9
		(0.3–5.5)		(0.3–5.4)

Data are medians and ranges. Concentrations of lipids in mmol/L are measured in plasma. Age is given in years. BMI: body mass index. SDS: standard deviation score. %BF: total body fat percentage. FFMI: fat free mass index. %TBF: truncal body fat percentage. TC: total cholesterol. LDL: low-density lipoprotein. HDL: high-density lipoprotein. TG: triglycerides.

### Associations between BMI SDS and body composition

**Fig 2:**
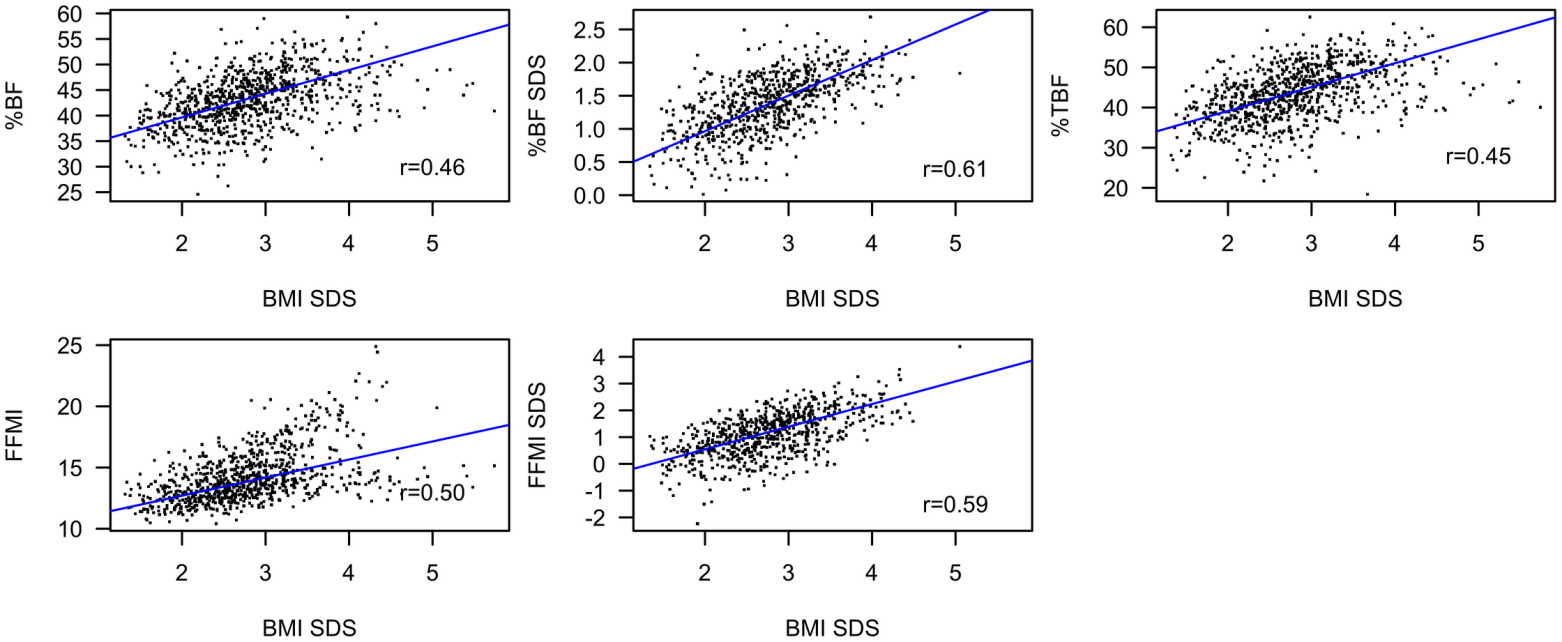
Baseline associations between BMI SDS and body composition variables. Plots of BMI SDS versus %BF SDS, %BF, %TBF, FFMI SDS, and FFMI at baseline. BMI: body mass index. SDS: standard deviation score. %BF: total body fat percentage. %TBF: truncal body fat percentage. FFMI: fat free mass index.

At baseline, BMI SDS was positively associated with %BF SDS (β = 0.36, 95% confidence interval (95%CI):[0.32;0.40], *p*<2*10^−16^), %BF (β = 3.96, 95%CI:[3.57;4.34], *p*<2*10^−16^), %TBF (β = 5.00, 95%CI:[4.53;5.46], *p* <2*10^−16^), FFMI SDS (β = 0.93, 95%CI:[0.86;1.00], *p* <2*10^−16^), and FFMI (β = 1.54, 95%CI:[1.43;1.66], *p*<2*10^−16^) (Fig 2).

**Fig 3:**
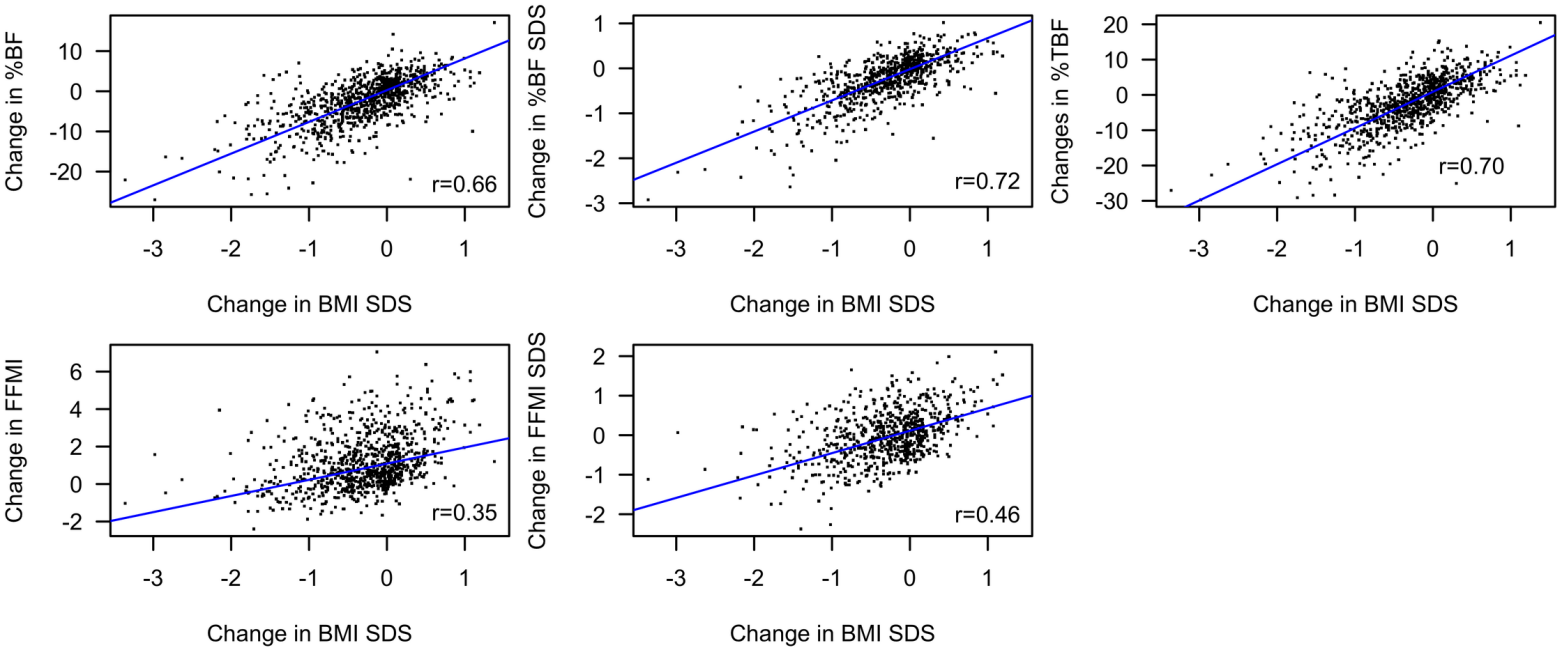
Associations between changes in BMI SDS and body composition variables. Plots showing the correlation between changes in BMI SDS and changes in %BF SDS, %BF, %TBF, FFMI SDS, and FFMI. BMI: body mass index. %BF: total body fat percentage. %TBF: truncal body fat percentage. FFMI: fat free mass index. SDS: standard deviation score.

Reductions in BMI SDS were associated with reductions in %BF SDS (β = 0.70, 95%CI:[0.66;0.74], *p*<2*10^−16^), %BF (β = 6.79, 95%CI:[6.39;7.18], *p*<2*10^−16^), %TBF (β = 8.93, 95%CI:[8.48;9.38], *p*<2*10^−16^), FFMI SDS (β = 0.51, 95%CI:[0.45;0.57], *p*<2*10^−16^), and FFMI (β = 0.93, 95%CI:[0.82;1.03], *p*<2*10^−16^) (Fig 3). Furthermore, the %BF SDS (95%CI[-0.6;-0.5], *p*>2.2*10^−16^), %BF (95%CI[-6.5;-5.2], *p*<2.2*10^−16^), FFMI SDS (95%CI[-0.6;-0.4], *p*<2.2*10^−16^), FFMI (95%CI[-1.0;-0.6], *p* = 3.8*10^−15^), and %TBF (95%CI[-8.6;-7.1], *p*<2.2*10^−16^) decreased more in the subgroup reducing their BMI SDS during treatment compared to the subgroup increasing their BMI SDS.

### Changes in BMI SDS, body composition indices and fasting plasma lipids during treatment

592 (68%) of 876 patients reduced their BMI SDS by a mean of 0.59 (95%CI:[0.55;0.63], *p*<2.2*10^−16^), 558/876 (64%) reduced their %BF by mean of 5.6% (95%CI:[5.2;6.0], *p*<2.2*10^−16^), 479/721 (66%) reduced their %BF SDS by a mean of 0.50 (95%CI[0.46;0.54], *p*<2.2*10^−16^), and 507 (58%) of 876 patients reduced their %TBF by a mean of 6.8% (95%CI:[6.3;7.3], *p*<2.2*10^−16^) during childhood obesity treatment for a median of 1.8 years (range 0.4–7.4 years) (Table 2). 82% of patients reducing either %BF, %BF SDS, or %TBF also reduced their BMI SDS. Patients who reduced their BMI SDS during treatment were more often girls (p = 2.6*10^−4^), were younger (p = 0.02), had a higher BMI SDS (p = 1.6*10^−5^) and a higher TG (p = 0.002) at baseline compared to the patients increasing their BMI SDS during treatment, but did not differ in any of the other baseline variables (p>0.05).

**Table 2:** Characteristics at baseline and follow-up in patients reducing or increasing their BMI SDS.

	Reduced BMI SDS				Increased BMI SDS			
	N	Baseline	N	Follow-up	N	Baseline	N	Follow-up
Sex	592	281/311			284	187/97		
(girls/boys)								
Age (years)	592	11.0	592	13.3	284	11.5	284	14.2
		(1.62–21.7)		(4.4–23.2)		(2.7–20.8)		(4.9–22.9)
Treatment	592	-	592	1.6	284	-	284	2.0
time (years)				(0.4–7.2)				(0.6–7.4)
BMI SDS	592	2.80	592	2.29	284	2.60	284	2.90
		(1.38–5.70)		(-0.49–5.2)		(1.32–4.40)		(1.42–4.70)
%BF SDS	492	1.40	568	1.10	229	1.40	271	1.50
		(0.17–2.70)		(-1.90–2.40)		(0.01–2.60)		(0.12–2.70)
FFMI SDS	492	1.10	568	1.00	229	1.10	271	1.40
		(-2.24–4.40)		(-1.79–3.9)		(-1.19–3.50)		(-0.68–3.20)
%BF	592	43.5	592	39.8	284	42.7	284	44.4
		(21.7–60.9)		(17.4–60.2)		(26.2–59.0)		(24.6–60.5)
FFMI	592	13.8	592	14.7	284	13.8	284	15.8
		(10.4–24.9)		(10.4–23.2)		(10.5–24.4)		(11.5–25.8)
%TBF	592	43.8	586	39.7	281	43.0	281	46.3
		(21.7–60.9)		(12.5–62.1)		(18.4–62.5)		(24.7–62.7)
TC (mmol/L)	325	3.9	267	3.7	144	4.0	113	4.0
		(1.7–5.9)		(1.7–6.3)		(2.3–6.5)		(2.5–7.8)
LDL (mmol/L)	324	2.3	267	2.0	142	2.3	112	2.3
		(0.6–4.4)		(0.5–2.3)		(1.0–4.6)		(0.8–4.2)
HDL (mmol/L)	325	1.2	267	1.3	144	1.1	113	1.2
		(0.6–2.3)		(0.6–2.3)		(0.6–2.0)		(0.6–2.0)
TG (mmol/L)	325	0.8	267	0.8	144	1.0	113	1.1
		(0.1–4.6)		(0.2–2.9)		(0.2–4.9)		(0.2–8.7)
Non-HDL (mmol/L)	325	2.7	267	2.4	144	2.8	113	2.8
		(0.7–4.7)		(0.7–4.8)		(1.2–5.5)		(0.9–6.9)
LDL/HDL	324	1.9	267	1.6	142	1.9	112	1.9
		(0.3–5.1)		(0.3–4.0)		(0.7–5.5)		(0.5–5.1)

Data are medians and ranges. Concentrations of lipids in mmol/L are measured in plasma. Age and treatment time are given in years. BMI: body mass index. SDS: standard deviation score. %BF: total body fat percentage. FFMI: fat free mass index. %TBF: truncal body fat percentage. TC: total cholesterol. LDL: low-density lipoprotein. HDL: high-density lipoprotein. TG: triglycerides.

In the 284/876 (32%) patients, who increased their BMI SDS by a mean of 0.29 BMI SDS (95%CI:[0.25;0.32], *p*<2.2*10^−16^), 79 (28%) decreased their %BF SDS, an additional 91 (32%) increased their FFMI SDS, and an additional 3 (1%) decreased their %TBF. Hence, 173/284 (61%) of those who increased their BMI SDS improved their body composition measured by DXA. Correspondingly, in the subgroup of 592 patients reducing their BMI SDS, 400 (68%) reduced their %BF SDS, an additional 18 (3%) increased their FFMI SDS, and further 60 patients (10%) reduced their %TBF. In total, 478/592 (81%) of the patients reducing their BMI SDS improved their body composition. Thus a significantly greater proportion of the patients reducing their BMI SDS improved their body composition (*p* = 5.5*10^−10^).

In the 301 patients with follow-up blood samples after a median of 1.5 years (range 0.4–7.2 years) (Fig 1), 81 (27%) increased their BMI SDS. Of these patients, 19 (23%) decreased their %BF SDS, an additional 29 (36%) increased their FFMI SDS, and an additional 2 (2%) reduced their %TBF. Furthermore, 64 (80%) of the 81 children who increased their BMI SDS improved one or more of their lipid concentrations. However, the patients reducing their BMI SDS did significantly improve their TC (95%CI[-0.5;-0.2], *p* = 1.5*10^−4^), LDL (95%CI[-0.3;0.0], *p* = 3.3*10^−4^), HDL (95%CI[0.0;0.1], *p* = 0.001), and TG (95%CI[-0.5;-0.2], *p* = 6.3*10^−7^) compared to patients increasing their BMI SDS. Patients with follow-up blood samples available did not differ from patients without follow-up blood samples available in regards to baseline age, %BF SDS, %BF, or the fasting plasma concentrations of TC, LDL, HDL, or TG, but the patients with follow-up blood samples had a higher baseline BMI SDS (*p* = 0.024) (S2 Table).

In total, 791 (90%) of the 876 patients either reduced their BMI SDS, reduced their %BF SDS or %TBF, increased their FFMI SDS, or improved one or more of their fasting plasma lipid concentrations.

### Associations between BMI SDS, body composition, and fasting plasma TC

Baseline BMI SDS was not associated with baseline TC (Table 3). Further adjusting the model for FFMI SDS (*p* = 0.02) or %BF SDS (*p* = 0.88) did not change this result. Neither baseline %BF SDS or %BF were associated with baseline TC, however, %TBF was positively associated with TC at baseline (Table 3).

**Table 3:** Associations between baseline BMI SDS, body composition indices and lipid concentrations in 469 children and adolescents with overweight/obesity.

	BMI SDS			%BF SDS			%BF			FFMI SDS			FFMI			%TBF		
	β	95%CI	*p*	β	95%CI	*p*	β	95%CI	*p*	β	95%CI	*p*	β	95%CI	*p*	β	95%CI	*p*
**TC**	0.07	(-0.04;0.18)	0.2	0.27	(0.06;0.48)	0.011	0.03	(0.01;0.04)	0.0019	-0.02	(-0.12;0.09)	0.75	-0.01	(-0.06;0.04)	0.71	0.02	(0.01;0.04)	3.46*10^**−4**^
**LDL**	0.07	(-0.02;0.17)	0.15	0.27	(0.09;0.44)	0.0031	0.03	(0.01;0.04)	4.6*10^**−4**^	-0.02	(-0.11;0.07)	0.64	-0.01	(-0.05;0.03)	0.71	0.02	(0.01;0.03)	1.34*10^**−4**^
**HDL**	-0.07	(-0.11;-0.03)	6.7*10^**−4**^	-0.03	(-0.11;0.04)	0.37	0.00	(-0.01;0.00)	0.36	-0.05	(-0.09;-0.02)	0.0041	-0.03	(-0.05;-0.02)	1.7*10^**−4**^	-0.01	(-0.01;0.00)	0.022
**Non-HDL**	0.14	(0.03;0.25)	0.012	0.31	(0.10;0.51)	0.0035	0.03	(0.01;0.04)	5.3*10^**−4**^	0.04	(-0.07;0.14)	0.48	0.02	(-0.02;0.07)	0.32	0.02	(0.02;0.04)	8.80*10^**−6**^
**LDL/HDL**	1.24	(1.09;1.42)	**0.0011**	1.43	(1.14;1.80)	0.0023	1.03	(1.01;1.05)	6.5*10^**−4**^	1.09	(0.97;1.22)	0.15	1.06	(1.00;1.12)	0.049	1.03	(0.02;0.04)	7.39*10^**−6**^
**TG**	1.2	(1.11;1.30)	9.7*10^**−6**^	1.17	(1.01;1.36)	0.038	1.01	(1.00;1.03)	0.016	1.11	(1.03;1.20)	0.0054	1.07	(1.03;1.11)	2.6*10^**−4**^	1.02	(1.01;1.03)	9.91*10^**−6**^

Estimates (β), 95% confidence intervals (95%CI) and p-values of the association between baseline body mass index (BMI) standard deviation score (SDS), total body fat percentage (%BF) SDS, %BF, truncal body fat percentage (%TBF), fat free mass index (FFMI) SDS, and FFMI and baseline fasting plasma lipid concentrations. Estimates for total cholesterol (TC), low-density lipoprotein cholesterol (LDL), high-density lipoprotein cholesterol (HDL), and non-HDL are in mmol/L. Estimates for triglycerides (TG) and LDL/HDL are in percentages due to logarithmic transformation. The data are linear regressions adjusted for baseline age, sex, and scanner type if appropriate. Significance level: *p*<0.0014. P-values meeting the significance threshold are marked with bold.

Reductions in BMI SDS were associated with reductions in TC (Table 4). The association persisted when adjusting for changes in FFMI SDS and baseline FFMI SDS (*p* = 2.1*10^−5^). However, additionally adjusting the model for the change in %BF SDS and baseline %BF SDS rendered the association insignificant (*p* = 0.96).

**Table 4:** Associations between changes in body composition and changes in fasting lipid concentrations in 301 children and youths with overweight/obesity.

	ΔBMI SDS			Δ%BF SDS			Δ%BF			ΔFFMI SDS			ΔFFMI			Δ%TBF		
	β	95%CI	*p*	β	95%CI	*p*	β	95%CI	*p*	β	95%CI	*p*	β	95%CI	*p*	β	95%CI	*p*
**ΔTC**	0.32	(0.20;0.44)	2.0*10^**−7**^	0.42	(0.29;0.55)	3.0*10^**−9**^	0.05	(0.04;0.06)	4.3*10^**−14**^	0.08	(-0.06;0.22)	0.25	0.05	(-0.02;0.12)	0.15	0.04	(0.03;0.05)	1.94*10^**−13**^
**ΔLDL**	0.28	(0.18;0.38)	8.6*10^**−8**^	0.38	(0.26;0.49)	3.1*10^**−10**^	0.04	(0.03;0.05)	3.1*10^**−15**^	0.06	(-0.05;0.18)	0.28	0.01	(-0.04;0.08)	0.51	0.03	(0.03;0.04)	3.29*10^**−14**^
**ΔHDL**	-0.13	(-0.17;-0.09)	2.1*10^**−10**^	-0.12	(-0.17;-0.07)	6.5*10^**−6**^	-0.01	(-0.01;-0.00)	1.4*10^**−4**^	-0.13	(-0.18;-0.08)	8.8*10^**−8**^	-0.06	(-0.09;-0.04)	2.4*10^**−8**^	-0.01	(-0.01;0.00)	9.95*10^**−5**^
**ΔNon-HDL**	0.44	(0.33;0.55)	1.1*10^**−13**^	0.52	(0.40;0.65)	2.3*10^**−14**^	0.05	(0.05;0.07)	<2*10^**−16**^	0.21	(0.07;0.34)	0.0027	0.11	(0.04;0.18)	0.0089	0.05	(0.04;0.06)	<2*10^**−16**^
**ΔLDL/HDL**	0.39	(0.28;0.50)	8.2*10^**−11**^	0.43	(0.31;0.54)	1.5*10^**−11**^	0.04	(0.03;0.06)	4.5*10^**−15**^	0.28	(0.17;0.40)	3.9*10^**−6**^	0.14	(0.08;0.20)	6.8*10^**−6**^	0.04	(0.03;0.04)	5.9*10^**−15**^
**ΔTG**	0.36	(0.25;0.47)	2.1*10^**−10**^	0.34	(0.20;0.48)	2.4*10^**−6**^	0.03	(0.02;0.04)	2.2*10^**−6**^	0.31	(0.18;0.44)	5.7*10^**−6**^	0.2	(0.14;0.26)	3.9*10^**−10**^	0.03	(0.02;0.04)	5.62*10^**−7**^

Estimates (β), 95% confidence intervals (95%CI) and p-values of the association between changes in body mass index (BMI) standard deviation score (SDS), total body fat percentage (%BF) SDS, %BF, truncal body fat percentage (%TBF), fat free mass index (FFMI) SDS, and FFMI and changes in fasting plasma lipid concentrations. Estimates for changes in total cholesterol (TC), low-density lipoprotein cholesterol (LDL), high-density lipoprotein cholesterol (HDL), non-HDL, and triglycerides (TG) are in mmol/L. The data are linear regressions adjusted for baseline values of the dependent variables, baseline age, treatment duration, sex, and scanner type if appropriate. Significance level: *p*<0.0014. P-values meeting the significance threshold are marked with bold.

### Associations between BMI SDS, body composition, and fasting plasma concentrations of LDL

Baseline BMI SDS was not associated with baseline LDL (Table 3). Adjusting for FFMI SDS (*p* = 0.007) or %BF SDS (*p* = 0.93) did not change this result. Baseline %BF and %TBF were positively associated with LDL at baseline (Table 3). Reductions in BMI SDS were associated with reductions in LDL (Table 4). The association persisted when adjusting for changes in FFMI SDS and baseline FFMI SDS (*p* = 1.7*10^−5^). However, adjusting for the change in %BF SDS and baseline %BF SDS rendered the association insignificant (*p* = 0.89).

### Associations between BMI SDS, body composition indices, and fasting plasma levels of non-HDL

Baseline BMI SDS was not associated with baseline non-HDL (Table 3). Adjusting for FFMI SDS (*p* = 0.002) or %BF SDS (*p* = 0.18) did not change this result. Baseline %BF and %TBF were positively associated with non-HDL at baseline (Table 3). Changes in BMI SDS were associated with changes in non-HDL (Table 4). The association persisted when adjusting for FFMI SDS (*p* = 2.3*10^−9^), but adjusting for %BF SDS rendered the association insignificant (*p* = 0.06).

### Associations between BMI SDS, body composition, and fasting plasma concentrations of HDL

Baseline BMI SDS was associated with baseline HDL (Table 3). This association persisted when adjusting for %BF SDS (*p* = 0.0011), but rendered insignificant when adjusting for FFMI SDS (*p* = 0.086). Neither %BF SDS, %BF nor %TBF were associated with HDL at baseline (Table 3). Changes in BMI SDS were inversely associated with changes in HDL (Table 4). The association persisted when adjusting for %BF SDS (*p* = 6.6*10^−8^) or FFMI SDS (*p* = 1.2*10^−6^).

### Associations between BMI SDS, body composition, and fasting plasma concentrations of TG

Baseline BMI SDS was associated with baseline TG (Table 3). This association persisted when adjusting for either %BF SDS (*p* = 1.3*10^−4^) or FFMI SDS (*p* = 9.1*10^−4^). %TBF was positively associated with TG at baseline (Table 3). Changes in BMI SDS were associated with changes in TG (Table 4). This association persisted when adjusting for %BF SDS (*p* = 6.1*10^−5^) or FFMI SDS (*p* = 5.6*10^−6^).

### Associations between BMI SDS, body composition, and LDL/HDL-ratio

Baseline BMI SDS was associated with the LDL/HDL-ratio (Table 3). This association persisted when adjusting for FFMI SDS (*p* = 5.3*10^−4^), but not when adjusting for %BF SDS (*p* = 0.027). Baseline %BF and %TBF were positively associated with LDL/HDL-ratio (Table 3). Changes in BMI SDS were associated with changes in LDL/HDL-ratio (Table 4). This association persisted when adjusting for %BF SDS (*p* = 5.1*10^−5^) or FFMI SDS (*p* = 2.3*10^−9^).

## Discussion

This study suggests that health benefits from childhood obesity treatment may exceed what is demonstrated solely by changes in BMI SDS. We found that reductions in BMI SDS were associated with desired improvements in body composition and in fasting plasma lipid concentrations. Still, of the patients increasing their BMI SDS, 61% improved their body composition. Furthermore, in the patients with follow-up blood samples available, 80% of patients increasing their BMI SDS improved one or more of their circulating plasma lipids. Thus, a thorough evaluation not exclusively based on BMI SDS, should be performed when evaluating the benefits of obesity treatment.

Consistent with existing literature [12,31–33], baseline values of BMI SDS were positively associated with baseline %BF, %BF SDS, %TBF, FFMI, and FFMI SDS. As fat free mass is negatively associated with all-cause mortality, and body fat is positively associated with all-cause mortality [34], an increase in fat free mass and reduction in fat mass appears to be the desired changes in body composition during weight loss, in order to positively impact all-cause mortality. In our study, 68% of the children and adolescents reduced their BMI SDS during a median of 1.8 years of treatment and a similar proportion reduced their %BF SDS. In addition, some patients who, based on their changes in BMI SDS, seemed not to benefit from the treatment, improved body composition and fasting plasma lipid concentrations, and thus their CVD risk. This is in line with a pilot study, showing improved lipid concentrations after a 12-week nutrition and exercise program, in spite of no apparent weight loss in 15 Latino adolescents (mean age 15 years) with obesity [35]. Our study outcome is also in line with another study of 53 American adolescents (29 males, mean age 12.1 years) with dyslipidemia and obesity, showing improvements in the degree of dyslipidemia, even in the absence of weight loss [36].

In accordance with our previous study of 240 Danish children and adolescents [14], we did not find any association between BMI SDS and fasting plasma concentrations of TC, LDL, and non-HDL at baseline. In the present study, we had the opportunity to investigate the elements of body composition, and their association with the different plasma lipid fractions. Although the associations did not meet the significance threshold for this study, there was a tendency towards a positive association between %BF SDS and %BF, and plasma concentrations of TC, LDL, non-HDL, LDL/HDL, and TG at baseline. This is in line with an American population study (N = 2 661, 1 424 males, aged 8–19 years) which reported a positive association between %BF and circulating levels of TC, LDL, and TG [37], but in contrast to a German study (N = 2 209, 1 112 males, age 3–16 years, BMI>90^th^ percentile) finding no significant association between %BF and LDL or TG [33]. However, in this study and in line with our study, BMI SDS was superior to %BF in predicting plasma levels of TG and HDL [33]. Supporting this notion, the association between BMI SDS and baseline plasma concentrations of HDL in our study seemed to be mediated by the association with FFMI SDS, as the association persisted when adjusting for %BF SDS, but was insignificant when adjusting for FFMI SDS.

Especially centrally stored fat has been directly associated with obesity related complications and increased cardiovascular risk [38–40]. In the present study, we investigated the %TBF as a measure of centrally deposited fat. At baseline we found positive associations between %TBF and TC, LDL, non-HDL, TG, and LDL/HDL-ratio indicating, that the centrally deposited fat may influence the development of dyslipidemia more than the total body fat. This method does, however, not distinguish between the subcutaneous and the visceral fat.

Changes in %BF SDS seemed to be the primary factor influencing changes in plasma concentrations of TC, LDL, and non-HDL, as the associations between changes in BMI SDS and changes in circulating levels of TC, LDL, or non-HDL were insignificant when adjusting for changes in %BF SDS. Changes in %BF SDS did not appear to explain all of the changes in HDL, TG, or LDL/HDL-ratio, as the changes in these variables were associated both with reductions in %BF SDS and FFMI SDS. Changes in %BF SDS, %BF, and %TBF correlated well with changes in BMI SDS. Changes in %TBF did not provide additional information about the changes in fasting plasma lipid concentrations compared to the changes in total body fat (%BF SDS and %BF).

During growth and development, profound changes occur in the body composition in both girls and boys [41,42]. As we wished to analyze changes in DXA indices across a wide age group of children, it was preferable to use SDS values, as they take the growth and development of children and adolescents into account. The ideal reference population would have been a Danish reference, but there are currently no Danish LMS references available for the entire age span and the requested measurements in our study. We estimated that the NHANES references for white girls and boys were the best available [19]. As the primary objective of the study was to investigate changes during treatment, any systematic error implied by using these references would be present both at baseline and follow-up, and each child or adolescent thereby serves as his/her own control. As the LMS data were only available from age eight years and older, we excluded we excluded patients under the age of eight in the analyses of %BF SDS and FFMI SDS. However, we performed the analyses with the entire group using the %BF and FFMI as absolute values as well, and adjusted these analyses for age. This approach did not change the conclusions drawn on the subgroup analyses using the SDS values.

As the present study represents a subgroup of the entire patient cohort, and has been chosen from the criterion of minimum two DXA scans performed, there is a possibility of selection bias. This might make our findings less generalizable to the entire patient population. However, since we observed a comparable therapeutic effect in this subgroup as reported in the entire patient cohort [28], we have no reason to believe that this subgroup analysis is biased. However, the present study does not report effects of the childhood obesity treatment *per se*, but rather describes the impact on body composition and fasting plasma concentrations of lipids, with concomitant changes in BMI SDS. As no control group was included in the present study, we do not know how the body composition variables or fasting lipid concentration would have acted without treatment. This is a limitation to the study.

Another limitation is that only 54% had blood samples drawn at baseline, and only 34% had blood samples drawn at entry *and* at follow-up. However, other than having a higher BMI SDS at baseline, patients with blood samples both at baseline and follow-up did not differ from the patients with only baseline blood samples in regards to baseline age, body composition, and fasting plasma lipid concentrations, indicating that they can be perceived as a representable subset. The patients without blood samples exhibited a longer treatment duration than the patients who had blood samples drawn at baseline. We sought to overcome the effect of treatment time by adjusting for this in the multiple regression models.

The DXA procedures and the blood sampling procedures changed during the study period. These are unfortunate conditions in the clinical practice at hospitals, however, relevant adjustments were made to correct for these changes, and we believe that the data before and after the procedure changed, are comparable.

A study in 92 healthy American adults (73% Caucasian) reported no significant differences in fat mass, lean tissue mass, total body mass, or %BF between the Lunar Prodigy and Lunar iDXA models. Compared with the Lunar Prodigy, an overestimation, primarily in the regional measurements in participants with obesity, was observed in iDXA results of the fat mass and %BF [43]. For the present study, we performed sensitivity analyses on the data on changes during treatment performed solely on the iDXA, which did not change the conclusions (S3 Table).

An internal quality control analyses did reveal some differences between the methods for blood analyses, and conversion factors were calculated to overcome these differences [21].

Pubertal developmental stage has been shown to affect the %BF in healthy Danish adolescents (N = 950, 536 males, age 7–15) [42], and the fasting circulating lipid concentrations in American adolescents with normal weight (N = 633, 317 males, age 8–18 years) [44]. In our study, information regarding pubertal developmental stage was only available in a subgroup, and only at baseline. All analyses were adjusted for age, although age ranges for pubertal stages can be wide [44].

No dietary records were obtained, but the blood samples were drawn after an overnight fast, and the fasting condition was confirmed by interview prior to the blood sampling. If the child or adolescent did not adhere to the fasting condition, a new appointment was made. Smoking and alcohol are known to affect the concentrations of lipids [45]. In our cohort, we had information of alcohol and smoking habits in 75%. Only 3% indicated smoking, and 7% indicated drinking alcohol, and only few did either on a regular basis. Therefore, we do not believe this to have influenced the results of our study.

The strengths of the study include the large number of children with a uniform ethnicity evaluated at baseline and follow-up with anthropometrics, DXA scans, and fasting plasma lipids.

In conclusion, this study showed improvements in body composition and in all the measured fasting plasma lipid fractions including non-HDL and LDL/HDL-ratio, the results of which are projected to improve CVD health [46,47]. Overall, our study outcome suggests that the applied multidisciplinary and multifaceted treatment of childhood obesity may reduce the CVD risk imposed by fasting dyslipidemia, even in absence of reductions in BMI SDS. Longitudinal studies are needed to elucidate if this effect persists, if it can be extrapolated to other obesity related complications, and whether these health promoting changes amount to decreased mortality later in life.

## Supporting information

S1 Table Example of a specific treatment plan for a child provided at the baseline visit. (DOCX)

S2 Table Overview of differences between different subgroups in the study. (DOCX)

S3 Table Sensitivity analyses on data solely from the iDXA. Associations between changes in body composition and changes in fasting lipid concentrations in 251 children and youths with overweight/obesity.
(DOCX)
