# Supplementary material for: Childhood obesity treatment; Effects on BMI SDS, body composition, and fasting plasma lipid concentrations
Source: PLoS One. 2018 Feb 14;13(2):e0190576. doi: 10.1371/journal.pone.0190576 (PMC5812566; doi:10.1371/journal.pone.0190576)
Supplement: S1 Table — (DOCX) [file pone.0190576.s001.docx]

**S1 Table:** **Example of a specific treatment plan for a child provided at the baseline visit**.

1. Breakfast every day.
2. NO high-calorie cereal.
3. Instead oatmeal with low fat milk (0.1-0.5% fat) or rye bread with cold cuts with max 6-9% fat or cheese max 13% fat.
4. Morning snack: NO wheat buns with chocolate for snack before lunch.
5. Instead rye bread with cold cuts with max 6-9% fat, vegetables (i.e. carrots), or fruit.
6. Lunch: NO sandwiches on wheat toast or high fat cold cuts or salad.
7. Lunch: Instead rye bread with cold cuts with 6-9% fat, i.e. fish, turkey, or chicken. Salad with max 1 tablespoon low fat dressing.
8. Afternoon snack: NO wheat buns with chocolate for snack before lunch.
9. Instead rye bread with cold cuts with max 6-9% fat, vegetables (i.e. carrots), or fruit.
10. Dinner: Use plate model with ½ plate vegetables, ¼ meats/fish, and ¼ potatoes, rice or pasta. Arrange the plate in the kitchen. Diet is ad libitum, but if a second serving is required, wait 20 minutes between each serving.
11. Eat vegetables every day.
12. Try 1 new vegetable each week.
13. Fast food max once every month.
14. Candy, cake, chocolate etc. max once per week and an amount of 10 DKR.
15. Drink water instead of sugar-enriched beverages.
16. Sugar and artificial sweetened beverages max 0.33 centiliters per week.
17. Max 2 pieces of fruit per day.
18. Screen time reduced to max 2 hours, and not prior to 5 PM.
19. Walk or ride bike to and from school every day.
20. Start to a sport, i.e. handball.
