# Supplementary material for: Childhood obesity treatment; Effects on BMI SDS, body composition, and fasting plasma lipid concentrations
Source: PLoS One. 2018 Feb 14;13(2):e0190576. doi: 10.1371/journal.pone.0190576 (PMC5812566; doi:10.1371/journal.pone.0190576)
Supplement: S2 Table — (DOCX) [file pone.0190576.s002.docx]

**S2 Table:** Overview of differences between different subgroups in the study.

|  | DXA only | Baseline blood samples and/or follow-up blood samples | | Baseline blood samples only | Follow-up blood samples | |
| --- | --- | --- | --- | --- | --- | --- |
|  |  |  | *p*_1_ |  |  | *p*_2_ |
| N | 407 | 469 |  | 168 | 301 |  |
| Sex (girls/boys) | 234/173 | 264/205 | 0.77 | 93/75 | 171/130 | 0.75 |
| Age | 11.0  (1.6−21.7) | 11.3  (4.0−20.8) | 0.22 | 11.6  (5.4−20.8) | 11.2  (4.0−20.3) | 0.32 |
| BMI SDS | 2.75  (1.32−5.74) | 2.76  (1.36−5.41) | 0.62 | 2.66  (1.38−5.21) | 2.82  (1.36−5.41) | 0.02 |
| %BF SDS | 1.38  (0.17−2.69) | 1.40  (0.01−2.44) | 0.81 | 1.37  (0.01−2.27) | 1.44  (0.08−2.44) | 0.15 |
| FFMI SDS | 1.16  (-2.24−4.39) | 1.10  (-1.51−3.15) | 0.25 | 1.03  (-1.51-3.03) | 1.17  (-1.19−3.15) | 0.53 |
| TC  (mmol/L) | - | 3.9  (1.7−6.5) | - | 3.9  (2.2−6.1) | 3.9  (1.7−6.5) | 0.91 |
| LDL  (mmol/L) | - | 2.3  (0.6−4.6) | - | 2.3  (0.8−4.4) | 2.3  (0.6−4.6) | 0.98 |
| HDL  (mmol/L) | - | 1.1  (0.6−2.3) | - | 1.1  (0.6−2.3) | 1.1  (0.6−2.2) | 0.67 |
| TG  (mmol/L) | - | 0.9  (0.1−4.9) | - | 0.9  (0.1−3.6) | 0.8  (0.1−4.9) | 0.93 |
| Treatment time  (years) | 2.2  (0.4−7.4) | 1.5  (0.4−7.2) | 1.7*10^-5^ | 1.5  (0.4−5.3) | 1.5  (0.6−7.2) | 0.12 |

*p*_1_ describes differences between baseline values in the patients with only DXA indices available, and the patients with both DXA indices and blood samples at baseline and/or follow-up available.

*p*_2_ describes differences between baseline variables in the patients with DXA and only baseline blood samples available compared to the patients with DXA and both baseline and follow-up blood samples available.

Differences in gender distribution investigated using Pearson’s Chi-squared test. Differences in numerical variables investigated using Wilcoxon signed rank test.
