# Supplementary material for: Childhood obesity treatment; Effects on BMI SDS, body composition, and fasting plasma lipid concentrations
Source: PLoS One. 2018 Feb 14;13(2):e0190576. doi: 10.1371/journal.pone.0190576 (PMC5812566; doi:10.1371/journal.pone.0190576)
Supplement: S3 Table — Associations between changes in body composition and changes in fasting lipid concentrations in 251 children and youths with overweight/obesity. (DOCX) [file pone.0190576.s003.docx]

**S3 Table: Sensitivity analyses on data solely from the iDXA:** Associations between changes in body composition and changes in fasting lipid concentrations in 251 children and youths with overweight/obesity.

|  | **ΔBMI SDS** | | | **Δ%BF SDS** | | | **Δ%BF** | | | **ΔFFMI SDS** | | | **ΔFFMI** | | | **Δ%TBF** | | |
| --- | --- | --- | --- | --- | --- | --- | --- | --- | --- | --- | --- | --- | --- | --- | --- | --- | --- | --- |
|  | β | 95%CI | *p* | β | 95%CI | *p* | β | 95%CI | *p* | β | 95%CI | *p* | β | 95%CI | *p* | β | 95%CI | *p* |
| **ΔTC** | 0.38 | (0.26;0.51) | **8.1*10^-9^** | 0.44 | (0.31;0.58) | **1.3*10^-9^** | 0.05 | (0.04;0.07) | **3.2*10^-13^** | 0.18 | (0.02;0.33) | 0.27 | 0.10 | (0.02;0.19) | 0.15 | 0.04 | (0.03;0.05) | **6.0*10^-13^** |
| **ΔLDL** | 0.33 | (0.22;0.44) | **6.0*10^-9^** | 0.40 | (0.29;0.51) | **6.2*10^-11^** | 0.05 | (0.04;0.06) | **1.2*10^-14^** | 0.12 | (0.00;0.26) | 0.28 | 0.06 | (-0.02;0.12) | 0.17 | 0.04 | (0.03;0.05) | **3.6*10^-14^** |
| **ΔHDL** | -0.11 | (-0.16;-0.07) | **1.2*10^-7^** | -0.11 | (-0.16;-0.06) | **1.4*10^-5^** | -0.01 | (-0.01;0.00) | **1.5*10^-4^** | -0.10 | (-0.16;-0.05) | **1.2*10^-4^** | -0.06 | (-0.09;-0.03) | **5.7*10^-6^** | -0.01 | (-0.01;0.00) | **1.1*10^-4^** |
| **ΔNon-HDL** | 0.49 | (0.37;0.61) | **3.3*10^-14^** | 0.55 | (0.42;0.68) | **1.4*10^-14^** | 0.06 | (0.05;0.07) | **<2*10^-16^** | 0.28 | (0.13;0.43) | **3.1*10^-4^** | 0.16 | (0.08;0.24) | **1.1*10^-4^** | 0.05 | (0.04;0.06) | **<2*10^-16^** |
| **ΔLDL/HDL** | 0.44 | (0.32;0.57) | **1.2*10^-11^** | 0.42 | (0.31;0.54) | **1.9*10^-11^** | 0.05 | (0.03;0.06) | **5.9*10^-14^** | 0.26 | (0.13;0.39) | **1.2*10^-4^** | 0.14 | (0.07;0.21) | **1.0*10^-4^** | 0.04 | (0.03;0.05) | **8.2*10^-14^** |
| **ΔTG** | 0.36 | (0.24;0.48) | **1.2*10^-8^** | 0.33 | (0.19;0.48) | **1.2*10^-6^** | 0.03 | (0.02;0.05) | **6.1*10^-6^** | 0.33 | (0.18;0.48) | **3.0*10^-5^** | 0.23 | (0.16;0.31) | **1.5*10^-9^** | 0.03 | (0.02;0.04) | **3.2*10^-6^** |

Estimates (β), 95% confidence intervals (95%CI) and p-values of the association between changes in body mass index (BMI) standard deviation score (SDS), total body fat percentage (%BF) SDS, %BF, truncal body fat percentage (%TBF), fat free mass index (FFMI) SDS, and FFMI and changes in fasting plasma lipid concentrations. Estimates for changes in total cholesterol (TC), low-density lipoprotein cholesterol (LDL), high-density lipoprotein cholesterol (HDL), non-HDL, and triglycerides (TG) are in mmol/L. The data are linear regressions adjusted for baseline values of the dependent variables, baseline age, treatment duration, and sex. Significance level: *p*<0.0014. P-values meeting the significance threshold are marked with bold.
